# Automated hippocampal sparing whole brain radiotherapy with simultaneous integrated boost for multiple brain metastases: Halcyon, HyperArc on TrueBeam, and coplanar TrueBeam

**DOI:** 10.1002/acm2.14570

**Published:** 2024-11-29

**Authors:** Shane McCarthy, Ryan Clark, Anthony Magliari, William St. Clair, Damodar Pokhrel

**Affiliations:** ^1^ Department of Radiation Medicine University of Kentucky Lexington Kentucky USA; ^2^ Varian Medical Systems Palo Alto California USA

**Keywords:** dosimetric scorecards, Halcyon, hippocampal sparing WB RT, HyperArc, multiple brain metastases, RapidPlan, simultaneous integrated boost, TrueBeam

## Abstract

**Purpose:**

To demonstrate the ease and feasibility that hippocampal sparing whole brain (WB) simultaneous integrated boost (HSWB‐SIB) plans can be generated using knowledge‐based planning and Eclipse Scripting Application Programming Interface (ESAPI) for three different modalities, HyperArc on TrueBeam (TB‐HA), a coplanar beam arrangement on TrueBeam (TB‐Co), and the ring‐mounted Halcyon LINAC (Hal).

**Methods:**

Twelve patients with 2–14 brain metastases were retrospectively replanned for HSWB‐SIB using a published HSWB RapidPlan model with modifications for the automated addition of SIB to metastases. Prescribed dose was 30 Gy to the WB planning target volume (PTV) and 50 Gy to the metastases in 10 fractions. Eclipse treatment planning system (v16.1) was used with a 6 MV‐FFF beam and Acuros XB dose algorithm.

**Results:**

The methodology was successfully used for all modalities, generating plans in under 30 min. The plan doses were normalized to the WB PTV *D*_95%_ receiving 30 Gy. Reporting values in the order of Hal, TB‐Co, and TB‐HA: The WB PTV received a *V*_48_ _Gy_ of 4.58, 3.98, and 4.45 cc with statistically insignificant differences (*p* = 0.806). The boost PTVs received a *D*_95%_ of 50.60, 50.43, and 51.13 Gy with statistically significant comparisons between TB‐HA and the other two modalities (*p* = 0.005). The hippocampus maximum dose was 11.81, 11.51, and 11.13 Gy with no statistically significant comparisons (*p* = 0.105). All other oragns‐at‐risk (OAR) doses were clinically acceptable. The modalities were evaluated using a dosimetric scorecard, achieving average scores of 84.85%, 86.45%, and 87.39%. End‐to‐end testing ensured the deliverability of the HSWB‐SIB plans for all modalities.

**Conclusion:**

The novel modification of the preexisting HSWB RapidPlan model with the automated inclusion of SIB objectives allows for easy, intuitive planning of complex HSWB‐SIB treatments. All modalities demonstrated can be used with clinically comparable results. Other institutions are recommended to pursue and validate this HSWB‐SIB technique to increase the accessibility of a single‐course of high‐quality treatment for patients with multiple brain lesions.

## 1 INTRODUCTION

Numerous studies have emphasized the significance and prevalence of brain metastases in radiation therapy as well as outlining the various techniques used to treat them, commonly claiming an occurrence rate of 20%–40% for patients with solid tumors developing brain metastases.^1^, ^2^, ^3^, ^4^, ^5^, ^6^, ^7^ Modern treatment of brain metastases often consists of a combination of surgical resection, whole brain (WB) radiotherapy or hippocampal sparing whole brain (HSWB) radiotherapy,^8^, ^9^, ^10^, ^11^, ^12^, ^13^ and some form of stereotactic radiosurgery or radiotherapy (SRS/SRT).^14^, ^15^, ^16^, ^17^ Numerous guidelines have been published with recommendations for these various techniques including NRG CC001^10^ for HSWB and the most recent RSS Practice Guidelines for brain SRS/SRT.^18^

It is very common for a patient to receive two separate courses of treatment, one treating the microscopic disease through a form of WB radiotherapy and a second course treating the macroscopic disease, the individual lesions, with SRS or SRT. Some studies have begun investigating combining these two separate courses into a single course, delivering a HSWB plan with a simultaneous integrated boost (SIB) to the lesions (HSWB‐SIB).^19^, ^20^, ^21^, ^22^, ^23^ Doing so would reduce the patient time in clinic, improve patient compliance and potentially improve clinical outcomes. However, these types of plans are complicated to create, often require experienced personnel, and a large time commitment for treatment planning.

Liu et al.^24^ demonstrated the usefulness of Varian's knowledge‐based planning RapidPlan models for standardizing and automating the delivery of complex HSWB plans, while achieving exceptionally low dose to the hippocampus. The HSWB model presented in this study was made readily available through Varian's Medical Affairs website. The model, however, was developed for HSWB, not including SIB.

Using the HSWB model as a starting point and modifying the implementation of the model in the Eclipse Treatment Planning Software (Varian Medical Systems, Palo Alto CA), would allow for the generation of a standardized, automated approach for creating HSWB‐SIB plans. The applicability of RapidPlan based HSWB‐SIB will be tested across multiple platforms and modalities: the ring‐mounted Halcyon LINAC (Hal) with its stacked and staggered multi‐leaf collimator (MLC); a coplanar beam arrangement on the TrueBeam LINAC with the standard Millenium 120 MLC; and using Varian's HyperArc module on the TrueBeam LINAC with the same M120 MLCs. This automation method could allow for the generation of complex radiotherapy plans, potentially shortening simulation to treatment time while providing a standardized and clinically effective solution for HSWB with SIB. By demonstrating the versatility of this novel method with multiple platforms, high‐quality HSWB‐SIB treatments in a single course of radiation therapy could be made available to more patients, including those near low‐resource clinics.

## 2 METHODS AND MATERIALS

### 2.1 Patient selection

Institutional review board approval was obtained to retrospectively replan 12 patients for HSWB‐SIB to multiple brain lesions. Selected patients had previously received some form of radiation therapy for multiple brain lesions, including WB, hippocampal‐sparing whole brain, and stereotactic radiosurgery/radiotherapy. All patients had physician‐delineated brain lesions and hippocampus contours using high‐resolution MRI images. Patients had 6.2 metastases on average, ranging from 2 to 14 with an average individual boost PTV volume of 1.76 cc. The closest metastasis was reported at 5.4 mm from the hippocampus, measured from PTV surface to hippocampus surface, with an average closest distance of 18.5 ± 10.8 mm. The average hippocampus volume was 3.63 ± 0.91 cc. The average brain volume was 1327 ± 140 cc. The average WB PTV volume was 1294 ± 142 cc, where WB PTV was defined as the brain minus the hippocampus avoidance zone (a 5 mm expansion from the hippocampi) and the PTV boost structures (Table 1).

**TABLE 1:** Summary of patient characteristics.

	Boost PTVs					
Plan	Amount	Volume (cc) *mean (range)*	Closest distance to hippocampus (mm)	Hippocampus volume (cc)	Brain volume (cc)	Brain‐PTV volume (cc)
**1**	14	0.64 (0.16–2.23)	17.8	2.40	1328	1292
**2**	8	0.94 (0.22–2.63)	17.6	4.76	1347	1316
**3**	11	1.31 (0.61–3.39)	6.1	4.83	1177	1136
**4**	4	0.94 (0.27–1.69)	27.9	3.61	1135	1106
**5**	3	0.90 (0.57–1.52)	15.7	3.25	1265	1243
**6**	2	6.40 (4.71–8.10)	47.5	4.46	1466	1438
**7**	6	0.22 (0.07–0.52)	24.3	3.58	1234	1208
**8**	6	3.45 (0.42–10.25)	14.2	4.17	1602	1577
**9**	6	1.68 (0.21–6.08)	11.1	4.33	1556	1525
**10**	5	1.51 (0.43–4.67)	13.9	3.67	1244	1214
**11**	6	1.49 (0.43–3.37)	5.4	1.99	1325	1294
**12**	3	10.45 (1.25–27.46)	21.0	2.45	1241	1184
**Average**	6.2 (2–14)	1.76 (0.07–27.46)	18.5 ± 10.8	3.63 ± 0.91	1327 ± 140	1294 ± 142

*Note*: Distance to hippocampus is measured from the surface of the hippocampus to the surface of the nearby PTV. Brain volume is the entire brain structure including lesions. Brain‐PTV volume is the aforementioned brain with the PTVs and hippocampus avoidance zone removed.

### 2.2 Planning techniques

#### 2.2.1 Dose prescription

The dose prescription used was 30 Gy in 10 fractions for the WB PTV with a simultaneous‐integrated boost of 50 Gy to the metastases. Plans were normalized such that 95% of the PTV WB received 30 Gy. The hippocampus was contoured following the guidelines established by Gondi et al.^25^ Dose constraints to OARs were adapted from the NRG CC001 protocol requirements for HSWB treatment^10^ as well as the QUANTEC guidelines.^26^ Plans were calculated using the Acuros XB dose calculation algorithm.^27^

#### 2.2.2 Beam geometries

The three modalities tested during this research were the coplanar Hal, a coplanar beam arrangement on TrueBeam (TB‐Co), and the noncoplanar HyperArc on TrueBeam (TB‐HA). The decision to utilize a TB‐Co, when noncoplanar beam arrangements have been shown to achieve higher plan quality for similar treatments,^28^ was to allow a more direct comparison with the coplanar Halcyon plan.

The Hal plans used four full arcs of a 6 MV‐FFF beam with collimator angles of 315°, 0°, 45°, and 90° alternating clockwise and counterclockwise gantry rotation. The Halcyon is equipped with a stacked and staggered MLC allowing for an effective leaf width of 0.5 cm at isocenter. The plan isocenter was aligned to the center of the WB PTV structure.

The TB‐Co plans used four full arcs of a 6 MV‐FFF beam as well, with collimator angles of 315°, 45°, and 90° with the latter angle being used twice for a superior/inferior beam split at the hippocampus. The TrueBeam is equipped with Millenium 120 MLC, with the 40 interior leaves having a 0.5 cm width at isocenter and the outer 20 having a 1 cm width at isocenter, for each bank. The plan isocenter was aligned to the center of the WB PTV structure, as done with the Hal plans.

The HyperArc plans used a full HyperArc beam arrangement with a 6 MV‐FFF beam, one full arc at a 0° couch angle and half arcs at 45°, 315°, and 270° couch angles. For each patient's unique tumor configuration, the collimator angles were optimized using the built‐in collimator optimization algorithm. Additionally, the plan isocenter was determined automatically by the HyperArc module and remained within the patient protection zone to enable automated delivery.

#### 2.2.3 RapidPlan with simultaneous integrated boost

Treatment planning was standardized and automated using Varian's RapidPlan in conjunction with Eclipse Scripting Application Programming Interface (ESAPI). As previously mentioned, Liu et al.^24^ demonstrated hippocampal sparing WB using a RapidPlan model that was made publicly available through Varian's Medical Affairs website.^29^ This model was acquired and validated for use. However, this model was trained on and used for HSWB, not including SIB, or intended for HyperArc geometry. Following dose volume histograms (DVH) Estimation, custom optimization objectives were injected using ESAPI to accommodate new boost PTVs and desired dose gradient location. New objectives for the boost target volumes as well as altering two of the automatically generated objectives from the RapidPlan model are presented in Table 2.

**TABLE 2:** Custom optimization objectives injected after DVH estimation to achieve the SIB distribution.

		Objectives			
Structure	Add/Remove	Type	Volume (%)	Dose (cGy)	Priority
Boost PTVs	Add	Lower	99.0	48.8	290
		Lower	100.0	44.5	270
		Upper	0.0	52.5	215
Boost GTVs	Add	Lower	100.0	51.5	200
		Upper	0.0	53.5	150
Hotspots	Add	Lower	100.0	52.5	250
		Upper	0.0	54.0	180
Rings	Add	Upper	0.0	52.5	180
PTV_WBopt07	Remove	Upper	0.1	31.5	300
		Upper	0.0	32.1	400
	Add	Upper	0.2	31.5	300
		Upper	0.1	32.1	400

*Note*: Additional optimization structures were used, their definitions can be found in the clinical document for the HSWB RapidPlan model.

Abbreviations: DVH, dose volume histograms; SIB, simultaneous integrated boost.

In addition to modifying and creating optimization objectives, new structures beyond those listed in the RapidPlan model's Clinical Description document were created. These modifications mostly involved subtracting the PTV boost volumes with an additional 5 mm margin from previous structures that now may result in conflicting objectives. Additionally, optimization structures consisting of a central sub volume of the GTVs and a surrounding ring structure facilitated a localized hotspot at the center of the GTV for each boost metastasis. The optimization structures modified and created are presented in Table 3.

**TABLE 3:** Modified and new optimization structures and their corresponding operations.

Structure	Operation
PTV_WBopt07	PTV_WB—(Hippo + 7 mm)—(PTV_Boost + 5 mm)
_Brainstem#Hi	Brainstem—(Hippo + 8 mm)—(PTV_Boost + 5 mm)
_Brain&BODY	(Brain + 20 mm)—(Brain + 3 mm)—(PTV_Boost + 5 mm) removed from outside BODY
Hippo05mm	Hippo + 5 mm—(PTV_Boost + 5 mm)
Hotspots	Inner 70% of GTVs
Rings	PTVs—Hotspots

Abbreviation: HSWB, hippocampal sparing whole brain.

**FIGURE 1:**
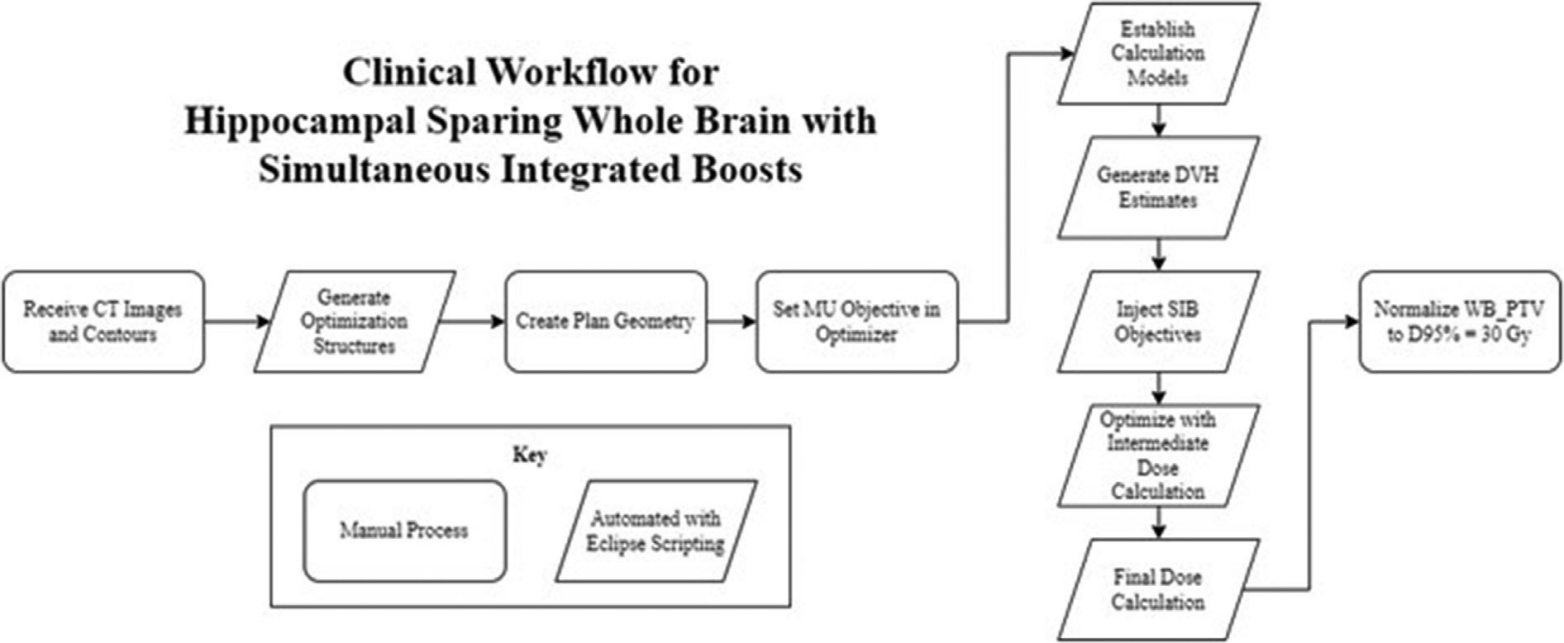
Flowchart outlining the clinical workflow for the HSWB‐SIB technique. As indicated by the key, steps outlined in a rhombus were automated using Eclipse Scripting, meaning no user interaction is required beyond starting the script. One exception is for creating plan geometry. If done using HyperArc, plan geometry is semi‐automated through the HyperArc module. If not using HyperArc, the plan geometry can be fully automated using Eclipse Scripting. HSWB‐SIB, hippocampal sparing whole brain‐simultaneous integrated boost.

Additional steps for the treatment planning process involved manually setting the MU objective in the optimizer, increasing the previous recommendation in the Clinical Description of the RapidPlan model to a minimum of 1600 MU and a maximum of 3500 MU with a priority of 80. Furthermore, the convergence mode calculation option for the Photon Optimizer (PO) was set to “On” instead of the recommended “Extended”, this change decreased the optimization time. During optimization a manual normaal tissue objectives (NTO) for the HSWBv2 model was used following the guidance of Clinical Description.^29^ Finally, the dose was normalized such that the WB PTV structure received 30 Gy to 95% of its volume. The final clinical workflow used is shown in Figure 1.

### 2.3 Plan evaluation

Metrics were used to assess this method's capability of achieving sufficient coverage to the WB PTV and the boost PTVs while minimizing dose to the hippocampus and other OARs. For the hippocampus, the minimum (*D*_100%_), mean, and maximum doses (*D*_0.03cc_) were evaluated. The maximum doses were also evaluated for the optic pathway, brainstem, spinal cord, lens, and skin. Additionally, the mean doses of the eyes were recorded.

For evaluating target coverage of the boost lesions, dose to 95% of the volume and minimum dose were evaluated for the PTVs. For GTVs, the minimum, mean, and maximum doses were recorded. With the addition of the SIB dose, the evaluation of the WB PTV became more involved. To begin, all plans were normalized such that the volume receiving 30 Gy was 95%. From there, the recommendation to evaluate the dose at 2% of the volume and the dose at 98% of the volume per NRG CC001 protocol, was followed. Due to the addition of the boost, a metric for the volume of the WB receiving 48 Gy was included. This metric is intended to quantify the boost dose near the lesions as well as assess the risk for brain radionecrosis.

In addition to the standard evaluation of dose metrics, a dosimetric scorecard was developed to further quantify plan quality. Dosimetric scorecards allow for comprehensive plan quality assessment and precise articulation of tradeoff preferences including the relative importance of competing OAR and target metrics. The scorecard was developed using the readily available, open source Dosimetric Scorecard tool provided through Varian's Medical Affairs website.^30^ The scorecard's metrics were based on the HSWBv2 RapidPlan model's scorecard,^29^ with slight modifications and additions to quantify the SIB portion of the plans.

**FIGURE 2:**
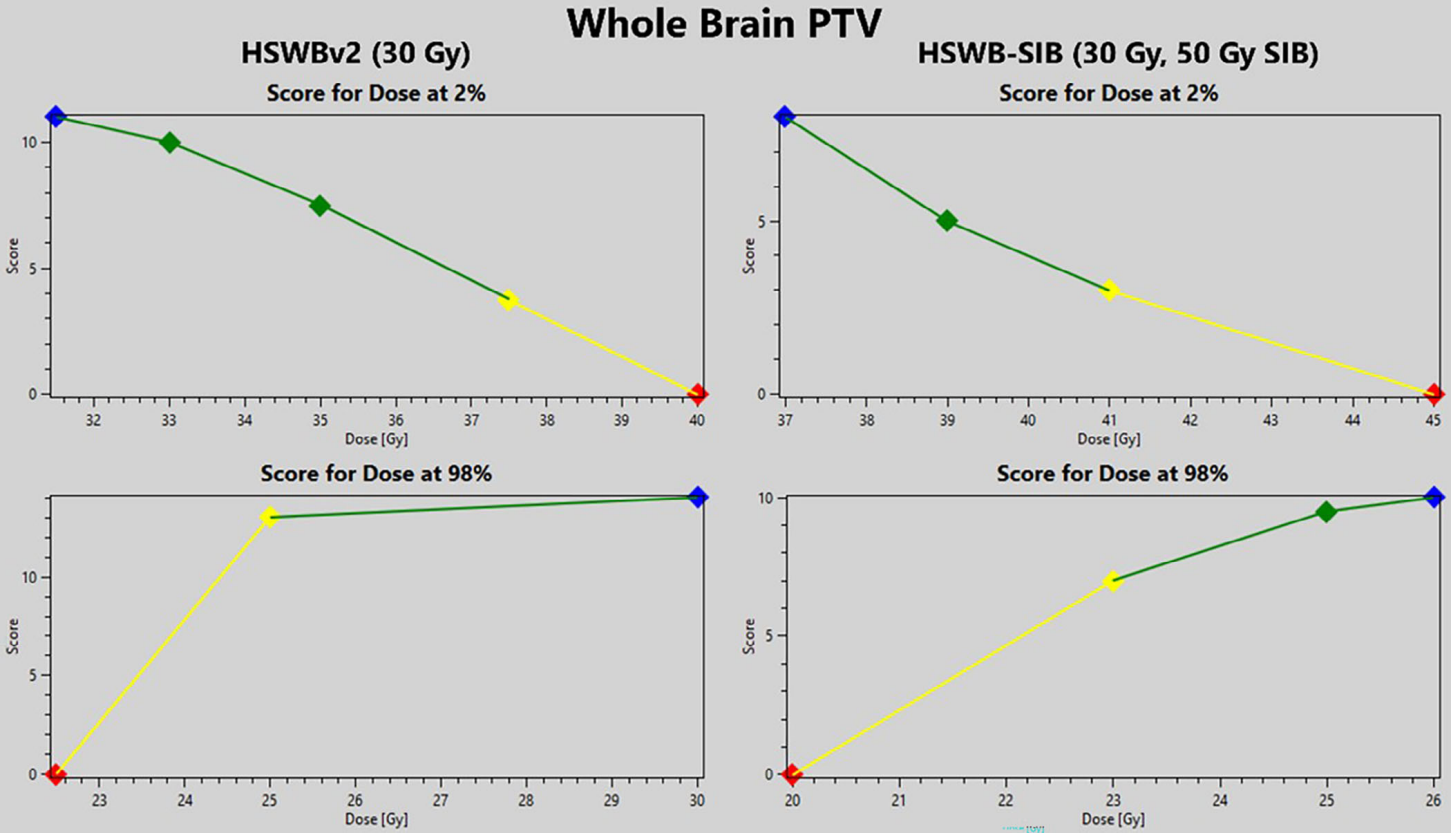
Piecewise linear functions provided from dosimetric scorecard tool for the WB PTV. The left graphs are for HSWBv2 Scorecard. The right graphs are for HSWB‐SIB Scorecard. Metrics shown are *D*_2%_ and *D*_98%_. The total possible scores are 142 and 182 points for HSWBv2 and HSWB‐SIB, respectively. HSWB‐SIB, hippocampal sparing whole brain‐simultaneous integrated boost; WB, whole brain.

Figure 2 shows a comparison between the HSWBv2 scorecard and the HSWB‐SIB scorecard for the *D*_2%_ and *D*_98%_ dose metrics of the WB PTV structure. The dosimetric scorecard determines the value for these metrics for each plan and then scores that metric using the piecewise linear functions shown above. The user can add as many, or as few, points to the piecewise linear function to shape the curve they feel is best suited for that metric. If a metric value falls above or below the defined points, the score given is the nearest defined point. The total possible number of points for a scorecard is determined by the user, the HSWBv2 scorecard has 142 total points while the HSWB‐SIB has 182 total points.

**FIGURE 3:**
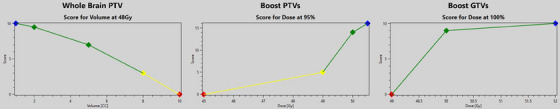
Piecewise linear functions provided from the dosimetric scorecard tool for the WB PTV *V*_48_ _Gy_, Boost PTVs *D*_95%_, and Boost GTVs *D*_100%_ from left to right, respectively. WB, whole brain.

For the SIB addition, the piecewise linear function for the high dose metric, *D*_2%_, for the WB PTV was extended to award points for up to 45 Gy, whereas the HSWBv2 scorecard would only award points for up to 40 Gy. Additionally, the maximum score for this metric was decreased for the HSWB‐SIB model, indicating this metric is not as valued as it was in the HSWBv2 model, however, additional metrics added to the HSWB‐SIB model also evaluate the high dose to the normal brain, offsetting this decrease in maximum score. Figure 3 shows three new piecewise linear functions added to the HSWB‐SIB model that were not in the HSWBv2 model. The volume receiving 48 Gy for the WB PTV was added to quantify the high dose received by the normal brain volume. This metric offset the decrease in maximum score to the *D*_2%_ for WB PTV. To elaborate, for the HSWBv2 model the *D*_2%_ metric could achieve a maximum score of 11, out of the total possible points of 142 this is roughly 8% of the total score is attributed to achieving an acceptable high dose to normal brain. The HSWB‐SIB scorecard the maximum score for the *D*_2%_ metric is 8, while the *V*_48_ _Gy_ metric can achieve a score of 10, in total 18 points out of the maximum score of 182, approximately 10% of the total score. This increase is to reflect the concern of high dose spill from the boost metastases. Figure 3 also shows the piecewise linear functions for the *D*_95%_ of the Boost PTVs and the *D*_100%_ of the Boost GTVs.

The modified scorecard was first developed around the inclusion of a single boost metastasis. This initial scorecard was then duplicated for additional metastases by dividing the total points for the boost metastases by the number of lesions. For instance, for a single boost metastasis, the *D*_95%_ metric for the Boost PTV can receive a maximum score of 16 points, for two boost metastases, each lesion can receive a maximum score of 8 points for this metric.

### 2.4 Quality assurance and independent dose verification

End‐to‐end testing was done to assess deliverability of the HSWB‐SIB plans using electronic portal imaging device (EPID)‐based portal dosimetry evaluated with a 3%/2 mm criteria.^31^ Independent dose verification was performed using an in‐house Monte Carlo (MC) second check software.^32^ Additionally, the total number of monitor units, modulation factor, beam on time, and total planning time were recorded. Total planning time was measured for the vertical portion of Figure 1.

### 2.5 Statistical analysis

After confirming the data's distribution was normal via the Shapiro‐Wilk test,^33^ an Analysis of Variance (ANOVA) test^34^ was performed. If the reported *p*‐value was below the significance threshold (*p* < 0.05), the null hypothesis was rejected and Tukey's test^35^ was performed to compare the mean of each sample to the mean of each other sample, determining which comparisons were statistically significant.

## 3 RESULTS

### 3.1 Target coverage

All plans were normalized such that 95% of the WB PTV received the WB prescription dose (i.e., *D*_95%_ = 30  Gy). Target coverage, as well as hippocampus dose, results can be found in Table 4. For the WB PTV, the high dose metrics, *D*_2%_ and *V*_48_ _Gy_, were comparable among the three modalities. The target coverage metric, *D*_98%_, reported statistical significance between TB‐HA and Hal (*p* = 0.008), indicating TB‐HA modality achieves better coverage of the WB PTV than the Hal modality.

**TABLE 4:** Evaluation of target coverage and hippocampus dose.

		Modality				
Structure	Metric	Hal	TB‐Co	TB‐HA	ANOVA *p*‐value	Significant comparisons
Hippocampus	*D*_100%_ (Gy)	6.39 ± 0.32 (5.96–7.10)	7.26 ± 0.29 (6.80–7.62)	7.65 ± 0.32 (7.27–8.23)	**< 0.001**	TB‐Co v TB‐HA TB‐Co v Hal TB‐HA v Hal
	*D*_mean_ (Gy)	8.56 ± 0.49 (7.88–9.32)	8.78 ± 0.39 (8.22–9.40)	8.73 ± 0.30 (8.23–9.23)	0.429	*‐None‐*
	*D*_0.03cc_ (Gy)	11.81 ± 0.96 (10.87–13.66)	11.51 ± 0.55 (10.63–12.33)	11.13 ± 0.61 (10.10–12.06)	0.105	*‐None‐*
Whole Brain PTV	*D*_98%_ (Gy)	27.90 ± 0.54 (26.57–28.58)	28.22 ± 0.46 (26.98–28.79)	28.57 ± 0.40 (27.69–29.08)	**0.008**	TB‐HA v Hal
	*D*_2%_ (Gy)	37.84 ± 3.34 (33.31–43.54)	37.13 ± 3.14 (33.26–42.18)	36.87 ± 2.94 (33.37–42.14)	0.755	*‐None‐*
	*V*_48_ _Gy_ (cc)	4.58 ± 2.63 (1.28–9.29)	3.98 ± 1.97 (1.28–7.29)	4.45 ± 2.21 (1.49–8.20)	0.806	*‐None‐*
Total Boost PTVs	*D*_95%_ (Gy)	50.60 ± 0.57 (49.42–51.64)	50.43 ± 0.41 (49.34–51.05)	51.13 ± 0.47 (50.33–51.94)	**0.005**	TB‐Co v TB‐HA TB‐HA v Hal
	*D*_100%_ (Gy)	46.14 ± 1.61 (43.27–48.59)	45.76 ± 1.82 (42.59–48.47)	47.13 ± 1.35 (44.50–49.16)	0.132	*‐None‐*
Total Boost GTVs (BED)	*D*_100%_ (Gy)	79.52 ± 2.07 (75.85–83.80)	79.19 ± 2.06 (74.24–81.70)	80.56 ± 1.73 (78.18–83.27)	0.248	*‐None‐*
	*D*_mean_ (Gy)	85.47 ± 1.81 (82.33–89.55)	84.82 ± 1.10 (83.26–87.18)	84.73 ± 1.34 (82.80–86.84)	0.434	*‐None‐*
	*D*_0.03cc_ (Gy)	88.65 ± 1.84 (85.93–92.42)	88.09 ± 1.29 (86.64–90.98)	87.34 ± 1.74 (84.44–89.80)	0.185	*‐None‐*

*Note*: Values are reported as mean ± standard deviation (range). A *p*‐value < 0.05 was considered significant. Dose values for the Total Boost GTVs are reported in BED with *α*/*β* = 10 Gy for tumor. Statistically significant *p*‐values are highlighted in bold.

Abbreviations: ANOVA, analysis of variance; BED, biological equivalent dose; Hal, Halcyon LINAC; TB‐Co, coplanar beam arrangement on TrueBeam; TB‐HA, HyperArc on TrueBeam.

Total boost PTVs’ *D*_95%_ showed statistical significance between the TB‐HA plans and the other two modalities (*p* = 0.005), suggesting slightly higher dose to tumors. The minimum dose to the total boost PTVs was comparable between the three modalities. Total boost GTVs metrics are reported in biological equivalent dose (BED), achieving an average BED mean dose across all three modalities of 85.01 ± 1.49 Gy. BED calculations were done using an alpha‐beta ratio of 10 Gy for the tumors as mentioned before. No statistical significance was found for any dose metrics reported for boost GTVs. The maximum dose of the GTVs remained below a physical dose of 60 Gy.

**FIGURE 4:**
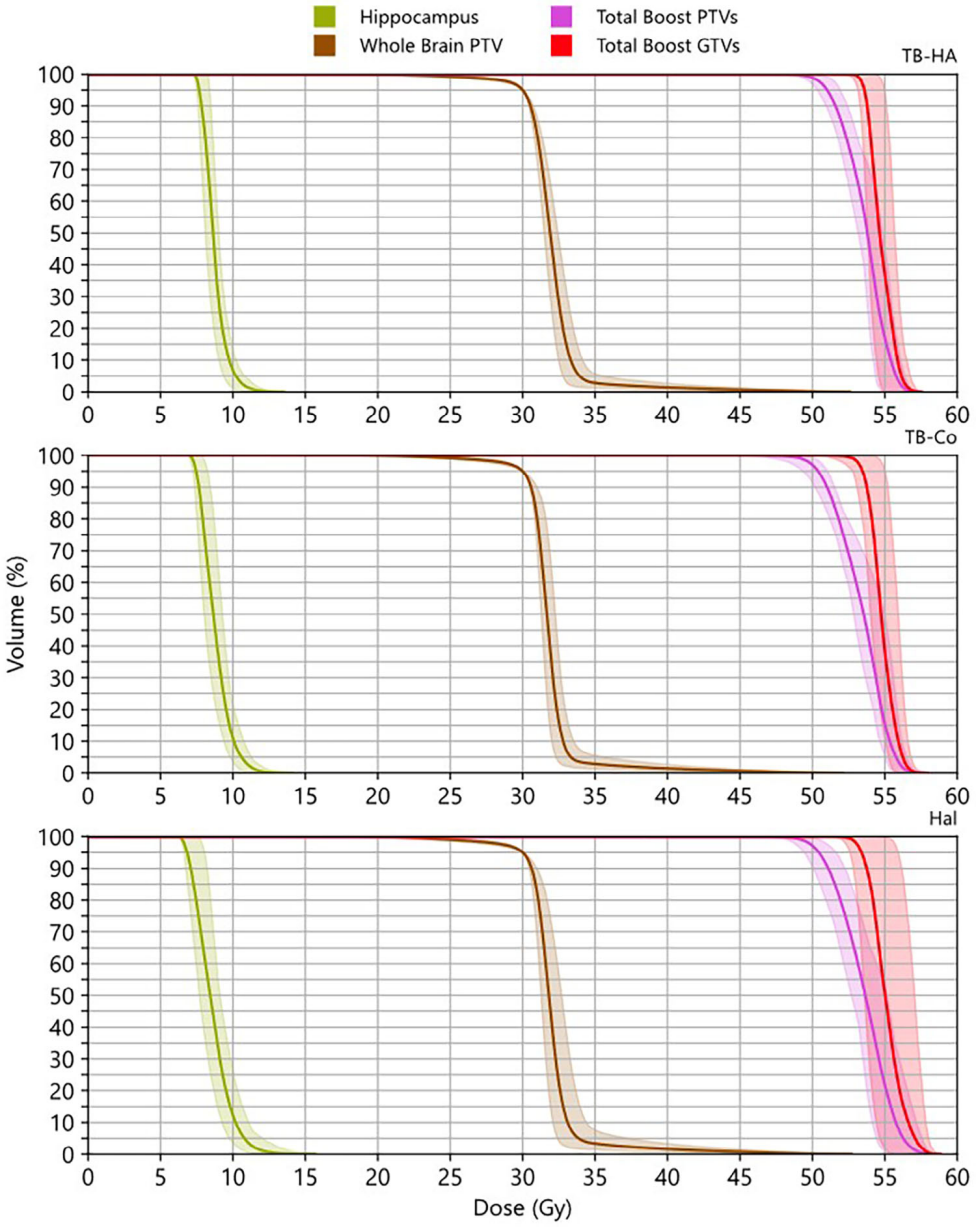
Composite DVHs for the three different modalities. The solid line is the mean value from all plans for that modality, the shadowed region spans from the minimum value to the maximum value from all plans for that modality. It is important to emphasize due to the context of this paper, these are not DVH estimation bands.DVHs, dose volume histograms.

Composite DVHs are presented in Figure 4, showing hippocampus, WB PTV, boost PTVs, and boost GTVs for the three different modalities. The graphs present the mean value (solid line) and the range (shadow). As shown on the graph, none of the modalities’ hippocampus maximum dose exceeds the 16 Gy criteria established by the NRG CC001 protocol. Boost PTV and GTV DVH ranges fall below common SRS/SRT prescription of the PTV *D*_95%_ receiving a prescription dose of 50 Gy in 10 fractions, for the TB‐Co and Hal plans, but not the TB‐HA plans. A sharper dose fall‐off for the boost lesions can be seen in TB‐HA plans.

**FIGURE 5:**
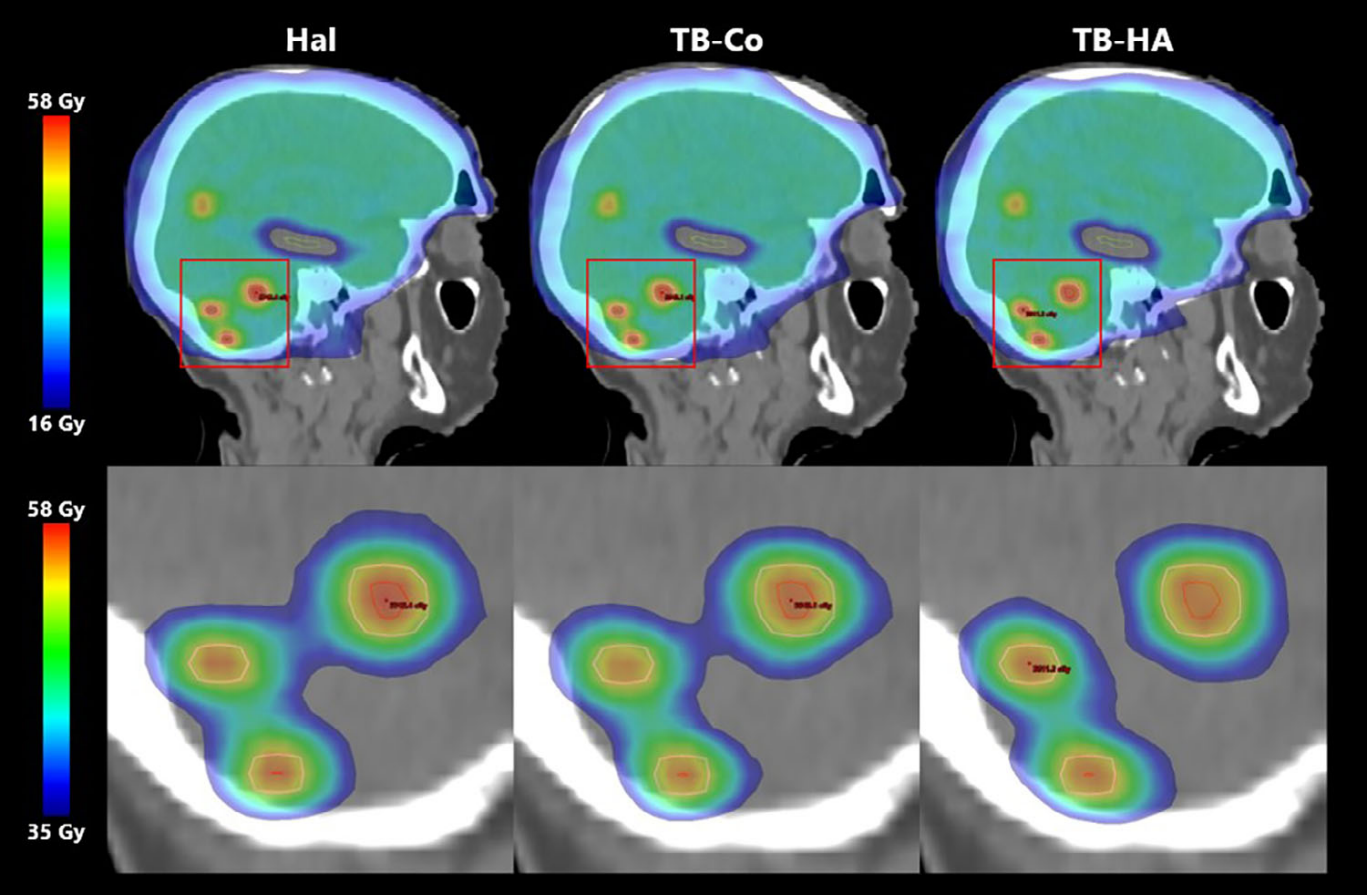
Isodose distributions for Case #1 with 14 metastases. The top row shows a sagittal view of the isodose colorwash for all three modalities, with a lower limit of 16 Gy on the dose colorwash. The bottom row shows a zoomed in view of the region of interest indicated in the above image, now with a lower limit of 35 Gy on the dose colorwash to better demonstrate the differences in conformity among the three modalities. Contours shown are GTVs in red, PTVs in pink, and hippocampus in green.

Figure 5 displays the isodose colorwash for Case #1 with 14 metastases for three different modalities. The top row displays a sagittal view focused on three lesions located in the inferior portion of the brain, with a fourth lesion beginning to appear slightly superior. Additionally, the hippocampus contour is nearby, with the dose colorwash lower limit set to 16 Gy indicating compliance with established protocol metrics. A magnified view of the three lesions, with the dose colorwash lower limit set to 35 Gy demonstrates the difference in conformality between modalities. As expected, TB‐HA approach allows slightly more conformal dose distributions, reducing the intermediate dose spread to brain tissue.

### 3.2 Dose to OARs

Shown in Table 4, the hippocampus received a statistically comparable maximum and mean doses throughout the three modalities. However, minimum dose was found to be statistically significantly lower for Hal than TB‐Co and TB‐HA as well as TB‐Co being statistically significantly lower than TB‐HA (*p* < 0.001).

In addition to reporting hippocampus sparing results, values for the brainstem, spinal cord, optic pathway structures, eyes, lens, and skin are shown in Table 5. For these values, the mean doses for the left and right eyes received statistically significantly (*p* = 0.010 and *p* = 0.002, respectively) lower doses for TB‐HA plans compared to the other modalities. Moreover, the right lens also achieved statistical significance (*p* = 0.002) in favor of the TB‐HA modality for maximum dose received. All other OARs received comparable doses.

**TABLE 5:** Reporting values for OAR doses. Mean ± standard deviation (range) was reported.

		Modality				
OAR	Metric	Hal	TB‐Co	TB‐HA	ANOVA *p*‐value	Significant comparisons
Brainstem	*D*_0.03cc_ (Gy)	35.23 ± 3.04 (32.83–41.84)	34.70 ± 2.94 (32.90–41.88)	34.68 ± 2.43 (32.61–40.38)	0.874	*‐None‐*
Spinal cord	*D*_0.03cc_ (Gy)	20.70 ± 5.58 (7.83–29.98)	20.64 ± 5.60 (8.31–29.69)	16.48 ± 6.70 (10.12–30.41)	0.181	*‐None‐*
Optic chiasm	*D*_0.03cc_ (Gy)	30.30 ± 0.57 (29.70–31.83)	30.15 ± 0.45 (29.61–31.13)	30.18 ± 0.48 (29.40–31.10)	0.759	*‐None‐*
Right optic nerve	*D*_0.03cc_ (Gy)	29.04 ± 1.45 (25.94–31.76)	29.02 ± 0.90 (27.56–31.02)	28.73 ± 2.27 (21.86–31.47)	0.889	*‐None‐*
Left optic nerve	*D*_0.03cc_ (Gy)	29.12 ± 0.73 (27.66–30.17)	28.78 ± 0.85 (26.77–30.12)	28.89 ± 1.48 (24.65–30.42)	0.751	*‐None‐*
Right eye	*D*_mean_ (Gy)	7.42 ± 1.14 (6.43–10.73)	7.30 ± 0.81 (6.59–9.65)	6.30 ± 0.61 (5.15–7.45)	**0.010**	TB‐Co v TB‐HA TB‐HA v Hal
Left eye	*D*_mean_ (Gy)	7.44 ± 0.98 (6.49–10.11)	7.33 ± 0.69 (6.45–8.91)	6.28 ± 0.66 (4.92–7.50)	**0.002**	TB‐Co v TB‐HA TB‐HA v Hal
Right lens	*D*_0.03cc_ (Gy)	4.94 ± 0.62 (4.07–6.15)	4.75 ± 0.57 (3.93–5.95)	4.02 ± 0.55 (2.90–5.25)	**0.002**	TB‐Co v TB‐HA TB‐HA v Hal
Left lens	*D*_0.03cc_ (Gy)	5.00 ± 0.69 (3.99–6.42)	4.73 ± 0.46 (4.17–5.69)	4.33 ± 0.71 (3.62–6.18)	0.057	*‐None‐*
Skin	*D*_0.03cc_ (Gy)	29.04 ± 0.56 (27.91–29.79)	28.70 ± 0.68 (27.83–30.42)	29.27 ± 0.68 (28.19–30.45)	0.128	*‐None‐*

*Note*: Statistically significant p‐values are highlighted in bold.

Abbreviations: ANOVA, analysis of variance; Hal, Halcyon LINAC; TB‐Co, coplanar beam arrangement on TrueBeam; TB‐HA, HyperArc on TrueBeam.

### 3.3 Dosimetric scorecard

The dosimetric scorecard evaluated various dose metrics of the plan and awarded points based on their values. The scorecard used for this research was developed to balance the WB PTV coverage against hippocampus sparing and reducing dose to OARs. Additionally, the scorecard evaluated target coverage for metastases. Multiple iterations of scorecard development were needed to ensure achieving a maximum total score is challenging. As a result, higher scores will always indicate a direction of potential improvement, albeit not always clinically achievable due to inherent tradeoffs.

The TB‐HA plans achieved the best scores with an average and standard deviation of 87.39 ± 5.49%. Following that, TB‐Co plans achieved 86.45 ± 4.97% and Hal plans achieved 84.85 ± 5.65%. The distributions of scores were similar and no statistical significance was measured. Table 6 reports the total score for various structures between the three modalities.

**TABLE 6:** Dosimetric scorecard scores for various structures across the three modalities.

		Modality				
Structure	Max Score	Hal	TB‐Co	TB‐HA	ANOVA *p*‐value	Significant comparisons
Whole Brain PTV	49	36.64 ± 6.88 (23.72–44.90)	38.19 ± 5.36 (28.21–44.73)	37.53 ± 5.71 (26.24–44.26)	0.832	*‐None‐*
Hippocampus	28	27.12 ± 0.61 (26.09–27.86)	26.83 ± 0.56 (25.90–27.64)	26.76 ± 0.52 (25.85–27.54)	0.288	*‐None‐*
Total boost PTVs	41	36.01 ± 6.79 (15.10–40.85)	36.51 ± 5.21 (21.09–40.71)	39.15 ± 2.13 (34.53–40.94)	0.312	*‐None‐*
Total boost GTVs	19	18.93 ± 0.15 (18.48–19.00)	18.78 ± 0.62 (16.75–19.00)	18.99 ± 0.03 (18.89–19.00)	0.419	*‐None‐*
Brainstem	3	1.51 ± 0.75 (0.00–2.21)	1.68 ± 0.76 (0.00–2.19)	1.62 ± 0.72 (0.00–2.29)	0.869	*‐None‐*
Spinal cord	3	3.00 ± 0.00 (3.00–3.00)	3.00 ± 0.00 (3.00–3.00)	2.99 ± 0.04 (2.86–3.00)	0.379	*‐None‐*
Optic chiasm	6	3.82 ± 1.70 (0.00–5.49)	4.09 ± 1.73 (0.00–5.39)	3.77 ± 1.89 (0.00–5.60)	0.903	*‐None‐*
Right optic nerve	6	4.95 ± 1.80 (0.00–6.00)	5.08 ± 1.80 (0.00–6.00)	5.05 ± 1.69 (0.00–6.00)	0.983	*‐None‐*
Left optic nerve	6	5.52 ± 0.53 (4.18–6.00)	5.75 ± 0.43 (4.45–6.00)	5.44 ± 0.88 (2.95–6.00)	0.498	*‐None‐*
Right eye	2	1.61 ± 0.24 (0.90–1.81)	1.63 ± 0.17 (1.13–1.78)	1.82 ± 0.10 (1.60–1.98)	**0.019**	TB‐HA v Hal
Left eye	2	1.60 ± 0.21 (1.04–1.80)	1.62 ± 0.14 (1.29–1.81)	1.81 ± 0.11 (1.59–2.00)	**0.006**	TB‐Co v TB‐HA TB‐HA v Hal
Right lens	2.5	1.84 ± 0.22 (1.41–2.15)	1.91 ± 0.20 (1.48–2.19)	2.16 ± 0.18 (1.73–2.50)	**0.002**	TB‐Co v TB‐HA TB‐HA v Hal
Left lens	2.5	1.82 ± 0.25 (1.31–2.17)	1.92 ± 0.16 (1.57–2.12)	2.05 ± 0.25 (1.39–2.30)	0.064	*‐None‐*
_Brainstem#HI	2	1.82 ± 0.15 (1.51–2.00)	1.91 ± 0.10 (1.74–2.00)	2.00 ± 0.00 (2.00–2.00)	**0.001**	TB‐HA v Hal
_Eyes&BODY	5	3.62 ± 0.80 (1.84–4.43)	3.66 ± 0.80 (1.81–4.57)	3.30 ± 0.54 (1.84–3.90)	0.453	*‐None‐*
_Brain&BODY	5	4.63 ± 0.34 (3.76–4.95)	4.78 ± 0.17 (4.39–4.98)	4.61 ± 0.32 (3.86–4.94)	0.342	*‐None‐*
Total score	182	154.43 ± 10.28 (133.43–168.69)	157.34 ± 9.04 (140.48–171.55)	159.04 ± 9.99 (142.30–171.45)	0.541	*‐None‐*

*Note*: Mean ± standard deviation (range) was reported. Statistically significant *p*‐values are highlighted in bold.

Abbreviations: ANOVA, analysis of variance; Hal, Halcyon LINAC; TB‐Co, coplanar beam arrangement on TrueBeam; TB‐HA, HyperArc on TrueBeam.

### 3.4 Planning feasibility, quality assurance, and deliverability

Results evaluating planning feasibility, quality assurance, and deliverability can be found in Table 7. The Hal and TB‐HA modalities delivered clinically acceptable plans validated by patient‐specific volumetric modulated arc therapy (VMAT) QA via EPID‐based portal dosimetry and independent MC 2nd check. For TB‐Co, one plan achieved a gamma passing rate of 89.5% for a threshold of 90%, beyond that all other TB‐Co plans were clinically acceptable. Statistical signficance was found for all parameters (*p* < 0.001).

**TABLE 7:** Planning feasibility, quality assurance, and deliverability metrics for all three modalities.

	Modality				
Parameter	Hal	TB‐Co	TB‐HA	ANOVA *p*‐value	Significant comparisons
Total calculation time (min)	23.01 ± 1.81 (19.38–26.25)	29.70 ± 5.63 (19.49–37.37)	27.47 ± 3.14 (21.50–33.08)	**0.001**	TB‐Co v Hal TB‐HA v Hal
Total monitor units	1909 ± 187 (1710–2345)	2710 ± 370 (2156–3507)	1899 ± 188 (1679–2235)	**< 0.001**	TB‐Co v TB‐HA TB‐Co v Hal
Modulation factor	3.82 ± 0.37 (3.42–4.69)	5.42 ± 0.74 (4.31–7.01)	3.80 ± 0.38 (3.36–4.47)	**< 0.001**	TB‐Co v TB‐HA TB‐Co v Hal
Beam‐on time (min)	2.39 ± 0.23 (2.14–2.93)	3.98 ± 0.01 (3.98–4.02)	2.50 ± 0.00 (2.50–2.51)	**< 0.001**	TB‐Co v TB‐HA TB‐Co v Hal
Patient‐specific VMAT QA *γ* pass rate (%)	99.99 ± 0.03 (99.90–100.00)	93.64 ± 2.52 (89.50–98.40)	97.51 ± 1.52 (94.40–99.30)	**< 0.001**	TB‐Co v TB‐HA TB‐Co v Hal TB‐HA v Hal
Monte Carlo 2nd check pass rate (%)	98.92 ± 0.64 (97.90–100.00)	97.05 ± 1.17 (94.80–99.10)	99.17 ± 1.24 (96.70–101.30)	**< 0.001**	TB‐Co v TB‐HA TB‐Co v Hal

*Note*: Values reported as mean ± standard deviation (range). Total calculation time evaluated during vertical portion of Figure 1. Patient‐specific VMAT QA uses a *γ* criteria of 3%/2 mm. Statistically significant *p*‐values are highlighted in bold.

Abbreviations: ANOVA, analysis of variance; Hal, Halcyon LINAC; TB‐Co, coplanar beam arrangement on TrueBeam, TB‐HA, HyperArc on TrueBeam.

Total calculation time, including plan optimization and calculating the final dose (see Figure 1), was found to be shortest for the Hal modality with a time of 23.01 ± 1.81 min. This value was statistically significant compared to 29.70 ± 5.63 min for TB‐Co and 27.47 ± 3.14 min for TB‐HA. It is important to note, photon optimizations and dose calculations were done with graphics processing unit (GPUs) enabled for the TB‐HA and Hal modalities, but not for TB‐Co. This is due to a known issue involving the combination of gEUD objectives and the GPU optimizer in PO v16.1.2. The gEUD objective is established by the RapidPlan model and is the same for all modalities, but this issue only occurs for the TB‐Co modality.

For the total number of monitor units, TB‐Co was found to have the highest with a mean and standard deviation of 2710 ± 370 MUs. This was statistically significant to the values for Hal and TB‐HA of 1909 ± 187 MUs and 1899 ± 188 MUs, respectively. A similar trend is seen in the modulation factor, a multiplicative of the total number of monitor units (see Table 7).

Beam‐on time was also found to be statistically significantly higher for TB‐Co, at 3.98 ± 0.01 minutes, compared to the other modalities at 2.39 ± 0.23 min for Hal and 2.50 ± 0.00 min for TB‐HA. TB‐HA value indicates this treatment modality is limited by gantry speed, not dose rate, considering the TrueBeam gantry speed of 1 rotation per minute with full HyperArc geometry consisting of one full arc and three partial arcs results in the mean value found of 2.5 min.

As previously mentioned, both Hal and TB‐HA had passing patient‐specific QA percentages for gamma analysis. TB‐Co, however, had one plan that achieved 89.5% for a 90% threshold. Statistical significance was found between each modality. Additionally, when compared to Eclipse AcurosXB calculation, MC 2nd check software reported clinically acceptable results for all modalities with a statistical significance between TB‐Co, 97.05 ± 1.17%, and the other two modalities, 98.92 ± 0.64% for Hal and 99.17 ± 1.24% for TB‐HA. Finally, all plans were found to be clinically acceptable by an experienced radiation oncologist.

## 4 DISCUSSION

This study highlights the accessibility and feasibility of highly complex HSWB‐SIB treatments by leveraging machine learning through knowledge‐based planning and automation with ESAPI. Automatically generated HSWB‐SIB plans achieved a conformal therapeutic dose to boost volumes while sufficiently sparing hippocampal structures and treating microscopic disease with a homogeneous distribution to the WB in a single course of radiation therapy. Moreover, the methodology was demonstrated across three different modalities, greatly increasing the accessibility of the treatment to underserved patient cohorts with limited access to resources.

Similar studies have been done in the past outlining potential benefits and feasibility of HSWB‐SIB treatments. Many of these studies limited the number of brain metastases being treated to less than eight brain metastases,^19^, ^23^ with some less than three brain metastases.^20^, ^22^ Additionally, these earlier manuscripts were published prior to NRG CC001 and demonstrated relatively limited hippocampal sparing. Popp et al.^8^ demonstrated HSWB‐SIB for 30 patients, achieving a hippocampal dose within agreement of NRG CC001, albeit at 12 fractions instead of 10 fractions. Due to the increased number of fractions, treating metastases to 51 Gy resulted in a target BED of 72.7 Gy as opposed to our target BED of 75 Gy for 50 Gy in 10 fractions (alpha‐beta ratio of 10 Gy for tumors). Finally, and most importantly, all previously mentioned studies required experienced planners spending several hours manually generating these treatment plans while the results presented in this study were generated in under 30 min, automatically, for three different modalities.

However, this study is not without limitations. The retrospective and single institution nature of the study limits the ability to fully demonstrate the robustness of this technique. Additionally, treating active cases is a necessary step going forward to fully implement this methodology into a clinic's workflow. Nevertheless, provided a diverse patient population with variation in tumor size, location, and occurrence, the method still demonstrated robustness. Injecting the same SIB objectives into the optimizer for all plans emphasized the accessibility and ease of such a technique, however, higher quality plans may be achieved with modality‐specific objectives. Additionally, selecting a convergence mode of “Extended” could potentially improve the quality of Halcyon plans, where additional leaf sequencing complexity is required due to the dual layer MLC. With only 12 patients, the statistical power of comparisons is not very strong. However, it is important to note that statistical significance is not the same as clinically significant; the absolute dose differences are relatively small among the modalities. Therefore, no claims are made as to which modality may be better than the other. Individual clinics are encouraged to test and use whichever method best suits their resources and needs.

Research continues to fully implement this method prospectively in our clinic and begin treating HSWB‐SIB patients. As hinted in the limitations, modality‐specific objectives are being developed as well as a user interface to allow planners to easily make informed modifications to objectives based on the current plan, similar to what has been done by Desai et al.^36^ Automating plan‐specific modifications would potentially decrease avoidable dose bridging between nearby lesions, improving conformity and reducing high to intermediate dose to normal brain. Additionally, an in‐depth analysis of the variation in patient setup accuracy and the resulting degraded composite conformality of high dose regions is underway to better assess the importance of a precise and reproducible patient setup. Further development of a generalized Simultaneous Integrated Boost Injector (SIBI) for RapidPlan could allow for injecting boost objectives into other pre‐existing RapidPlan models to treat other sites with SIB dose, such as prostate stereotactic body radiation therapy.^37^

## 5 CONCLUSION

A novel method was introduced to modify the use of preexisting RapidPlan models with the inclusion of SIB objectives to generate clinically acceptable HSWB‐SIB plans in under 30 min for three different modalities. The workflow established in this study enables less skilled treatment planners to generate high quality plans with ease and efficiency. These plans achieve a therapeutic dose for the macroscopic disease while adequately covering the WB PTV and effectively treating the microscopic disease. Furthermore, these plans excel at sparing the hippocampus within tolerance of the NRG CC001 requirements. Implementing the aforementioned methodology would potentially enable busier clinics to use their time more effectively, while allowing smaller, more remote, clinics to provide the same quality of care to a commonly underserved patient cohort.

## AUTHOR CONTRIBUTIONS

D.P. and S.M. conceptualize this clinical project. S.M. modified the model, collected, and analyzed the data. D.P., R.C., and A.M. provided their expertise to fine tune the model. W.S. provided radiation oncology clinical expertise and supervision of the paper. S.M. and D.P. prepared the preliminary manuscript. All co‐authors revised, edited, and approved the final manuscript for submission.

## CONFLICT OF INTEREST STATEMENT

All co‐authors have no conflict of interest to declare.

## Data Availability

No data available on request due to privacy/ethical restrictions.
